# Impact of COVID-19 on medical education and the challenges: how prepared is Nigeria?

**DOI:** 10.11604/pamj.supp.2020.37.1.24915

**Published:** 2020-12-14

**Authors:** Edmund Ndudi Ossai

**Affiliations:** 1 Department of Community Medicine, College of Health Sciences, Ebonyi State University Abakaliki, Abakaliki, Nigeria,; 2 Department of Community Medicine, Alex Ekwueme Federal University Teaching Hospital, Abakaliki, Nigeria

**Keywords:** COVID-19, medical education, Nigeria

## Abstract

Nigeria has one of the largest concentration of human resources for health in Africa. There are 46 accredited medical schools and the majority are owned by the various State Governments. The COVID-19 outbreak was declared a global pandemic on the 11^th^ of March 2020 by the World Health Organization. The Federal Government of Nigeria through the Federal Ministry of Education closed all tertiary institutions in Nigeria including the medical schools on the 19^th^ of March 2020 so as to curtail the effects of the pandemic. The effect of COVID-19 pandemic on tertiary institutions in Nigeria include the disruption of the academic calendar of the schools. This is capable of affecting the mental health of medical students. The smooth financing of medical education in Nigeria could be at risk. The career progression of medical students and those in the Residency Training Program and the doctors seeking greener pastures abroad are all affected. Unfortunately the state of infrastructure in the medical schools could be said to be poor. However, a good medical education scheme is said to guarantee the medical security of the populace. The Government and its agencies should work out plans of ameliorating the effects of the pandemic on medical education. This could also be a period to re-position the sector so as to be able to face similar challenges in future. The time has come for the full application of technology in delivering medical education in Nigeria. Incidentally, the pandemic has encouraged the application of e-learning techniques for the continuing professional development of medical doctors in the country. This should be the new way to go.

## Commentary

Nigeria based on the number of essential health workers has one of the largest concentration of human resources for health in Africa [1]. Incidentally, it is one of the 57 countries that has critical shortage of health workers. These countries have low densities of nurses, midwives and doctors when compared to the developed countries hence designated as ineffective in delivering essential services to their citizens. In effect, the health system of Nigeria is regarded as being weak. The country has a total of 46 accredited medical schools. Eighteen of these schools are owned by the Federal Government of Nigeria while the various state governments are in charge of twenty medical schools. Currently eight medical schools in Nigeria are privately owned [2]. The country is also served by two postgraduate medical colleges, the National Postgraduate Medical College of Nigeria and the West African Postgraduate Medical College that comprises the West African College of Physicians and West African College of Surgeons. The Medical and Dental Council of Nigeria (MDCN) and the Nigerian Universities Commission (NUC) are the government agencies that regulate medical education in Nigeria. The activities of the NUC is centered around academic standards while the MDCN focuses on the quality of the medical graduates from the various medical schools [3]. In Nigeria, medical doctors are trained at the undergraduate level and this runs for a period of six years. The first year of training is regarded as the preliminary year, the second and third years belong to the pre-clinical years while the fourth to sixth year is regarded as clinical period of training. The students commence medical internship immediately after graduation and could proceed for their postgraduate medical training after completing the one year mandatory National Youth Service Corps scheme. The doctors are expected to have passed the primary fellowship examination of any of the postgraduate medical colleges before commencing the Residency Training Program.

**COVID-19 outbreak:** the COVID-19 outbreak was declared a global pandemic on the 11^th^ of March 2020 by the World Health Organization. The disease was first reported in Wuhan city, China in December 2019 and is caused by a newly discovered coronavirus, SARS-CoV-2. Globally, the economic impact of COVID-19 is estimated to be the highest since the financial crisis of 2008/09 and has been described as having the capacity to affect the ability of government, households and development partners to fund education. Perhaps, based on its capacity to restrict international travels coupled with the imposition of national lockdowns in quick succession in several countries, the virus has been described as being contagious both medically and economically. Thus, it has been affirmed that the COVID-19 pandemic could spread economic suffering globally. Already the International Monetary Fund is of the opinion that with the effect of the pandemic, the global economy will contract by 3% in 2020. It has been projected that the pandemic is capable of causing Africa to experience its first recession in 25 years after two decades of economic progress with the effect that a third of jobs in Africa have been estimated to be in jeopardy. As at 3^rd^ July 2020, the world has recorded a total number of 10,662,536 confirmed cases of COVID-19 and 516,209 deaths [4].

**Impact on medical education:** with the outbreak of COVID-19, the Federal Government of Nigeria through the Federal Ministry of Education announced the closure of all tertiary institutions in Nigeria including the medical schools on the 19^th^of March 2020. This was seen as a way of curtailing the spread of the virus. Academic activities in the various state and private universities in the country were also brought to a halt. The effect of COVID-19 on tertiary institutions in Nigeria is expected to cause amongst others the disruption of academic calendar, teaching and learning gaps and a reduction in budgetary allocation to education [5]. Inadequate funding has already been identified as the key challenge to the delivery of medical education in Nigeria [6]. Suffice it to say that medical education in Nigeria is highly subsidized by the Government. The continued support to the universities in Nigeria may prove very difficult at this point in time due to dwindling resources as a result of the fall in the prize of crude oil which is Nigeria's major source of revenue. The Federal Government of Nigeria has highlighted two main responses to the COVID-19 pandemic which is based on saving lives of Nigerians and the preservation of the livelihood of workers and business owners. In this regard, the government put a halt to the planned sack of workers in the banking sector by their employers. This may be a good relief as the lockdowns occasioned by the pandemic has also adversely affected the economy of individuals and households. In any case the loss of jobs post-COVID in Nigeria is also a possibility. However in the 2020 revised national budget that has been passed by the national assembly, the budgetary allocation to Health and Education were reduced. These are the two ministries that are directly involved in providing medical education in Nigeria.

There has been no clear cut statement from the Federal Ministry of Education, the Committee of Vice Chancellors of Nigerian Universities and the Provosts of Medical Colleges on the way forward for medical education in Nigeria in the face of COVID-19 pandemic. Also, bearing in mind that the various State Governments in Nigeria control most of medical schools in Nigeria, (20 out of 46) the possibility of a pay cut for the medical teachers or outright non-payment of salaries may arise. This if implemented will have far reaching effects on the future of medical education in Nigeria. Unlike the Federal Government of Nigeria, not all the states in the federation pay salaries of workers promptly. Some State Universities in Nigeria are presently not admitting medical students because of their inability to meet the requirements of Medical and Dental Council of Nigeria for re-accreditation of their medical degree program. The continued disruption of the academic calendar in the medical schools will be of tremendous effect. Such disruptions of academic calendar have almost become a routine in higher institutions in Nigeria with the frequent industrial actions usually embarked by trade unions in the sector especially the Academic Staff Union of Universities. There could also be the psychological impact of the delay on the students especially those in the final year of study or those preparing to write any of the main examinations in medical school. This is capable of affecting the mental health of the students. The delay in graduation will also affect the recruitment of next batch of medical interns in Nigeria. Unfortunately many newly graduated doctors do not immediately commence their medical internship because of the non-availability of spaces in the specified health facilities accredited for such programs. So the pandemic will further delay the commencement of medical internship by the final year students. Already the two postgraduate medical colleges have postponed the first set of 2020 postgraduate medical examinations (April/May 2020), for their candidates in Nigeria due to the COVID-19 pandemic. Thus the Residency Training Program in Nigeria is already being affected. A similar postponement also took place in the country during the ebola epidemic of 2014. The Continued Professional Development (CPD) programs for medical doctors practicing in the country have also been disrupted. Attendance to these CPD courses is now a pre-requisite for the renewal of annual practicing license of physicians in Nigeria. Similarly, all the scientific conferences of various professional groups in the country have been cancelled since the COVID-19 period. Thus this may affect the professional development of practicing physicians in Nigeria.

It may affect the immediate plans of some qualified medical doctors who are in the process of securing or have secured job opportunities outside the country as the pandemic has restricted movements in and out of Nigeria due to the lockdowns. In fact such arrangements have been placed on hold by the receiving countries since the COVID-19 pandemic. The pandemic is also capable of affecting the career progression of newly qualified medical doctors who may have planned to pursue their specialist medical training outside Nigeria. A study among medical students in southeast Nigeria revealed that an approximate one third of the students intend to pursue specialist medical training outside Nigeria [7]. In a similar study among qualified medical doctors practicing in Nigeria, it was found that majority of the doctors preferred job opportunities abroad [8]. It remains to be seen how the COVID-19 pandemic will affect the emigration of medical doctors from the developing countries to the developed ones. There has been a call that the Government of Nigeria should increase the funding to higher institutions so as to enable them cope with the damages caused by the closure of schools due to the pandemic [5]. It will be recalled that during the Severe Acute Respiratory Syndrome (SARS) epidemic, some medical schools in China cancelled formal teaching in wards. However, one medical school initiated online problem based learning techniques to ensure that the curricula was completed. This became useful years after the epidemic [9]. In effect, the use of technologies have been identified of being capable of providing infrastructure and basis for taking care of the several challenges in providing medical education in the future.

It has been suggested that e-learning techniques and Information and Communications Technology should be taught in medical schools in Nigeria. These measures were perceived as meaningful tools for self-instructions and life-long learnings [6]. In a study among medical students in a Nigerian medical school, majority of the students were dissatisfied with the state of infrastructure in the university and wished for improvement [10]. The current pandemic has disrupted in-class instructions in the medical schools and it is uncertain when the situation will return to normal. Also, the clinical training of medical students no longer hold due to the unpredictability of the virus coupled with the non-availability of personal protective equipment in the various teaching hospitals. Moreover, there could be a possibility of such disruptions in the future. The authorities of the various medical schools in Nigeria should endeavor to lay the foundation for good response by their various institutions should a challenge of this nature present in the future. Consequently, there has been a call to the Nigerian Government to integrate all higher institutions in Nigeria into online education [5]. This has become pertinent now.

## Conclusion

It has been postulated that a good medical education guarantees medical security of the populace [6]. This necessitates that the Government and its agencies like the Medical and Dental Council of Nigeria and other stakeholders in the sector should work out plans of ameliorating the effects of the pandemic on medical education. This could also be a period to re-position the sector so as to be able to face similar challenges in future. Already the pandemic has encouraged the application of e-learning techniques for the Continuing Professional Development of medical doctors in Nigeria. This development is very new and should be sustained and improved upon. The application of technology to medical education in Nigeria is now of utmost relevance. The time to act is now.
